# Implementation of evidence-based practice across medical, nursing, pharmacological and allied healthcare professionals: a questionnaire survey in nationwide hospital settings

**DOI:** 10.1186/1748-5908-8-112

**Published:** 2013-09-24

**Authors:** Yi-Hao Weng, Ken N Kuo, Chun-Yuh Yang, Heng-Lien Lo, Chiehfeng Chen, Ya-Wen Chiu

**Affiliations:** 1 Department of Pediatrics, Chang Gung Memorial Hospital, Chang Gung University College of Medicine, Taipei, Taiwan; 2 Center for Evidence-Based Medicine, Taipei Medical University, Taipei, Taiwan; 3 Division of Preventive Medicine and Health Services Research, Institute of Population Health Sciences, National Health Research Institutes, Miaoli, Taiwan; 4 Department of Public Health, Kaohsiung Medical University, Kaohsiung, Taiwan; 5 Division of Plastic Surgery, Department of Surgery, Wan Fang Hospital, Taipei Medical University, Taipei, Taiwan; 6 Evidence-Based Medicine Center, Wan Fang Hospital, Taipei Medical University, Taipei, Taiwan; 7 Department of Public Health, School of Medicine, College of Medicine, Taipei Medical University, Taipei, Taiwan; 8 Master Program in Global Health and Development, College of Public Health and Nutrition, Taipei Medical University, 250 Wu-Hsing Street, Taipei, 110, Taiwan; 9 Health Policy and Care Research Center, Taipei Medical University, Taipei, Taiwan

## Abstract

**Background:**

Implementation of evidence-based practice (EBP) is regarded as core competence to improve healthcare quality. In the current study, we investigated the EBP of six groups of professionals: physicians, nurses, pharmacists, physical therapists, technicians, and other allied healthcare personnel.

**Methods:**

A structured questionnaire survey of regional hospitals throughout Taiwan was conducted by post in 2011. Questionnaires were mailed to all healthcare workers of 11 randomly selected hospitals. Linear and logistic regression models were used to examine predictors for implementing EBP.

**Results:**

In total, 6,160 returned questionnaires, including 645 from physicians, 4,206 from nurses, 430 from pharmacists, 179 from physical therapists, 537 from technicians, and 163 from other allied healthcare professionals, were valid for the analysis. Physicians and pharmacists were more aware of EBP than were the other professional groups (p < 0.001). Positive attitudes toward and beliefs in EBP were significantly lower among nurses than in the other groups (p < 0.001). Physicians had more sufficient knowledge and skills of EBP than did the other professionals (p < 0.001); in addition, they implemented EBP for clinical decision-making more often and perceived fewer personal barriers to EBP (p < 0.001). Multivariate logistic regression analyses showed that EBP implementation was associated with the following characteristics of participants: EBP training, having a faculty position, academic degree, one's profession, and perceptions (beliefs, attitudes, knowledge, skills and barriers).

**Conclusions:**

This study depicts various levels of EBP implementation among medical, nursing, pharmacological, and allied healthcare personnel. There were significant differences in their implementation of EBP. We observed that certain factors were associated with EBP implementation, including personal backgrounds and perceptions toward EBP. The data suggest that strategies for enhancing EBP implementation should differ for various groups of professionals.

## Background

Evidence-based practice (EBP) is clinical practice consistent with the current best evidence [1]. Implementation of EBP mainly involves four sequential steps [2]: first, framing a clear question based on a clinical problem; second, searching for relevant evidence in the literature; third, critically appraising the validity of contemporary research; and fourth, applying the findings to clinical decision-making. There are increasing examples illustrating that EBP can help healthcare professionals improve care quality [3-5]. Implementing EBP by all health professionals is thus needed [6-10].

Numerous studies have investigated the perceptions of EBP among a variety of healthcare-related professional groups [9-17]. Overall, most healthcare professionals hold positive attitudes toward EBP but lack sufficient knowledge and skills for implementation. A number of personal and organizational barriers impede EBP implementation [9,16-22]. To date, investigations of how health professionals implement EBP in clinical decision-making are still lacking [23,24].

In regional hospitals of Taiwan, the majority of disease patterns are more complex than in primary-care settings. Health providers in regional hospitals devote most of their time to patient care. Therefore, it is essential to promote EBP among regional hospitals in order to improve the quality of healthcare. Since the beginning of 2007, the National Health Research Institutes (NHRI) has provided EBP-related information resources and promotional activities for healthcare professionals of regional hospitals in Taiwan [25]. Despite the NHRI’s considerable ongoing efforts to encourage implementation of EBP, diffusion into regional hospitals is not yet widespread.

The backgrounds of physicians, nurses, pharmacists, and allied healthcare professionals naturally differ. Little research has focused on comparing the use of EBP among different healthcare professions. For EBP to be fully implemented, it is essential to clarify possible differences among professions. In this survey, we systematically assessed how EBP is perceived among all groups of healthcare professionals. This nationwide study allowed us to compare and contrast various levels of awareness, beliefs, attitudes, knowledge, skills, barriers, and implementation among various professions. The data provide critical evidence that can be used to guide strategies for improving the effectiveness of EBP dissemination.

## Materials/methods

### Design

A structured questionnaire was developed by the NHRI using questions based on our previously reported questionnaires [16,26]. The study was conducted during the four-month period of January to April 2011.

### Subjects

Targets of this study were healthcare professionals working in regional hospitals in Taiwan. A regional hospital is defined as a secondary-care hospital accredited by Taiwan’s Joint Commission of Hospital Accreditation. Cluster sampling was used to conduct the present study. Briefly, regional hospitals were divided into four clusters by location (northern, western, eastern and southern Taiwan), and a random sample of each cluster was selected. Since there were more hospitals in northern and western Taiwan, we selected more hospitals in those areas. Overall, we randomly enrolled 11 of the 65 regional hospitals in Taiwan, including 3 located in northern Taiwan, 4 in western Taiwan, and 2 each in eastern and southern Taiwan. The postal questionnaires were distributed to all healthcare professionals at the enrolled hospitals.

### Questionnaire

The questionnaire included items for measuring the awareness of, beliefs in, attitudes toward, knowledge of, skills in, barriers to, and implementation of EBP. Questions were as follows.

1. Awareness: Have you heard of EBP (evidence-based practice) or related terms, such as EBM (evidence-based medicine), EBN (evidence-based nursing), or EBHC (evidence-based healthcare)?

2. Beliefs: Do you believe EBP is important for improving patient care quality?

3. Attitudes: Are you willing to support the promotion of EBP implementation?

4. Knowledge: Do you have sufficient knowledge to implement EBP principles?

5. Skills: Do you possess sufficient skills to implement EBP principles?

6. Implementation: In the past year, have you searched for relevant evidence in the literature to resolve your clinical questions, and then applied the findings to clinical decision-making after critical appraisal?

A. Have you changed your clinical decision-making through EBP implementation?

B. Have you added new clinical decision-making through EBP implementation?

C. Have you confirmed your clinical decision-making through EBP implementation?

Questions were rated using a Likert 5-point scale (strongly agree, agree, neutral, disagree, and strongly disagree) for attitudes, knowledge, skills and barriers.

Background characteristics included gender, age, teaching appointment, administrative position (defined as healthcare professionals who are in charge of administrative affairs), working experience, and academic degree. Academic level was divided into five categories: technical school degree; junior college (two-year university) degree; bachelor’s degree (which requires seven years for medical school, six years for dental school, and four years for the other specialties); master’s degree; and doctorate.

### Validity and reliability

The content validity was examined by 10 experts with more than 15 years of clinical experience each [25]. The internal consistency of all indexes was estimated using Cronbach’s alpha coefficient [25]. In this survey, a content validity index of 0.96 and Cronbach’s coefficient alpha of 0.88 indicated sufficient validity and reliability of parameters in the questionnaire.

### Ethical considerations

NHRI’s Ethical Review Board approved the study protocol. The questionnaire was accompanied by an introductory letter stating the purpose of the study and promising confidentiality. Return of the completed questionnaire was considered consent to participate in the study. All questionnaires were anonymous.

### Statistical analyses

The Likert 5-point scale was dichotomized for further analyses. A self-rating report of either ‘strongly agree’ or ‘agree’ was regarded as a favorable answer, while the other three (‘neutral,’ ‘disagree,’ and ‘strongly disagree’) were viewed as unfavorable answers. The statistical analyses were conducted using SPSS 12.0 for Windows (SPSS, Chicago, IL, USA). Categorical variables were analyzed using a Chi-square test. To explore predictors for the self-reported implementation of EBP, multiple logistic regression models were used. All odds ratios (ORs) and 95% confidence intervals (CIs) were adjusted for the other variables. Significance was defined as p < 0.05.

## Results

### Awareness of EBP

In total, 10,770 questionnaires were distributed to healthcare professionals of the enrolled hospitals, with 6,360 questionnaires returned (for a return rate of 59.1%). Of these, 6,160 valid questionnaires, 645 were from physicians, 4,206 from nurses, 430 from pharmacists, 179 from physical therapists (including occupational therapists), 537 from technicians (including diagnostic medical sonographers, medical technologists, and radiographers), and 163 from other allied healthcare professionals (including 5 from speech language therapists, 13 from psychological consultants, 50 from respiratory therapists, 67 from dietitians, and 28 from other groups of personnel).

Among the 6,160 respondents, 5,038 were aware of EBP or related terms (81.8%). Awareness of EBP was associated with the following personal characteristics: gender (p < 0.001), age (p < 0.001), working experience (p < 0.001), academic degree (p < 0.001), a faculty position (p < 0.001), a directorial position (p < 0.001), and one's profession (p < 0.001) (Table 1). The greatest awareness of EBP was among physicians (95.2%), and technicians (95.2%), followed by pharmacists (93.3%), other allied professions (84.7%), physical therapists (83.2%), nurses (79.7%).

**Table 1:** Awareness of evidence-based practice (EBP)

	**All respondents**	**Aware of EBP**	**p-value**
	**N = 6,160**	**N = 5,038 (%)**	
**Gender**			<0.001
Male	942	815 (86.5%)	
Female	5,218	4,223 (80.9%)	
**Age (years)**			<0.001
20 ~ 30	2,760	2,083 (75.5%)	
31 ~ 40	2,571	2,211 (86.0%)	
41 ~ 50	664	599 (90.2%)	
> 50	165	145 (87.9%)	
**Working experience (years)**			<0.001
< 5	2,108	1,611 (76.4%)	
5 ~ 10	2,384	1,934 (81.1%)	
> 10	1,668	1,493 (89.5%)	
**Academic level**			<0.001
Technical school	1,846	1,384 (75.0%)	
Junior college	1,809	1,464 (80.9%)	
Bachelor’s*	2,062	1,773 (86.0%)	
Master’s	401	377 (94.0%)	
Doctorate	42	40 (95.2%)	
**Faculty position (%)**			<0.001
Yes	1,219	1,129 (92.6%)	
No	4,941	3,909 (79.1%)	
**Director (%)**			<0.001
With	620	597 (96.3%)	
Without	5,540	4,441 (80.2%)	
**Profession**			<0.001
Physician	645	614 (95.2%)	
Nurse	4,206	3,354 (79.7%)	
Pharmacist	430	401 (93.3%)	
Physical therapist	179	149 (83.2%)	
Technician	537	382 (71.1%)	
Other	163	138 (84.7%)	

* The bachelor’s curriculum is seven years for medical school, six years for dental school, and four years for the other specialties.

### Beliefs in, attitudes toward, knowledge of, and skills of participants

**Figure 1:**
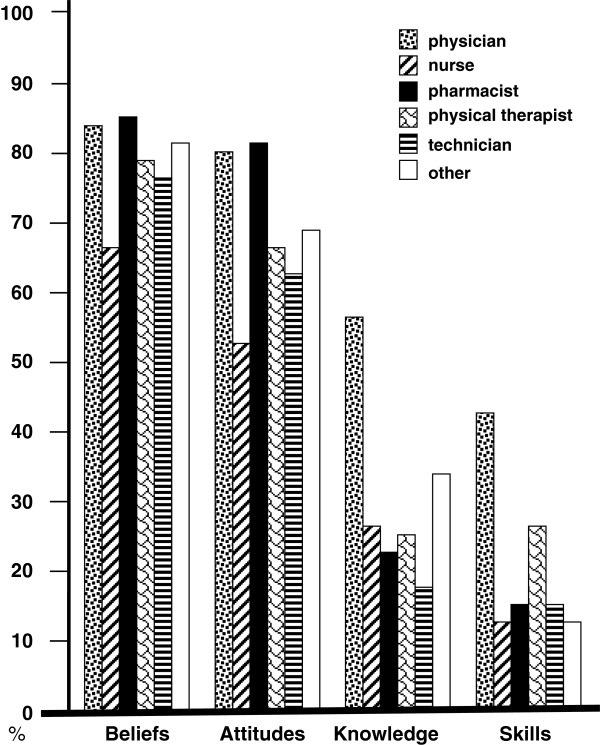
**Four evidence-based practice (EBP) characteristics among 6 groups of healthcare professionals.** Beliefs: EBP is important to improve patient care quality. Attitudes: I am willing to support the promotion of EBP implementation. Knowledge: I have sufficient knowledge to implement EBP principles. Skills: I possess sufficient skills to implement EBP principles.

Among the 5,038 participants who were aware of EBP, 3,604 healthcare professionals reported believing that EBP is important for improving patient care quality (71.5%), and 3,015 stated that they were willing to support implementation of EBP (59.8%) (Figure 1). However, their self-reported knowledge of (28.7%) and skills in (16.8%) implementing EBP principles were relatively insufficient. There were significant discrepancies in beliefs, attitudes, knowledge and skills among the six groups of professions (p < 0.001). Physicians and pharmacists were more likely to hold positive beliefs in and attitudes toward EBP (p < 0.001). Furthermore, physicians tended to have more sufficient knowledge of and skills in EBP than did the other professional groups (p < 0.001).

### Implementation of EBP

**Figure 2:**
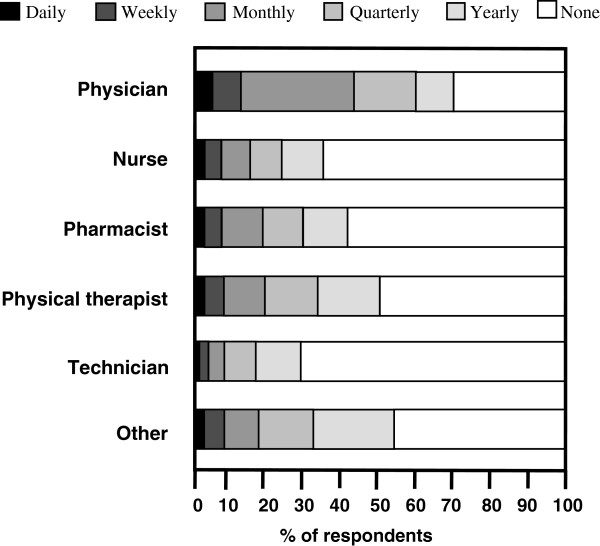
Frequency of evidence-based practice implementation for clinical decision-making among six groups of healthcare professionals.

Among the 5,038 participants who were aware of EBP, 2,111 respondents reported having implemented EBP for clinical decision-making in the previous year (41.9%). There were significant differences in the frequency of EBP implementation among the six groups of professionals (p < 0.001) (Figure 2). Physicians reported implementing EBP the most (p < 0.001). In contrast, nurses, pharmacists, and technicians reported implementing EBP the least. Overall, 166 reported implementing EBP daily (7.9%), 266 weekly (12.6%), 568 monthly (26.9%), 480 quarterly (22.7%), and 631 yearly (29.9%).

**Figure 3:**
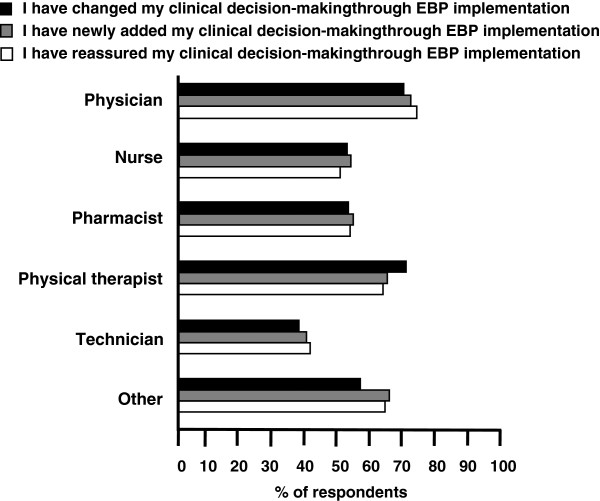
Behavior of evidence-based practice (EBP) implementation for clinical decision-making among six groups of healthcare professionals.

The behaviors reported for EBP implementation involved changes in (57.0%), additions to (58.4%), and reaffirmations of (56.8%) decision-making (Figure 3). Physicians and physical therapists more often implemented EBP to change, add and reaffirm their decision-making, whereas technicians implemented EBP the least (p < 0.001). However, there were no statistically significant differences in these three behaviors within any professional group.

### Barriers to and training in EBP

Barriers to implementing EBP were categorized into personal and environmental factors. Table 2 summarizes the barriers of personal and environmental factors. Barriers in relation to the organizations and colleagues of the respondents were regarded as environmental factors. The most common barrier was a lack of convenient kits (such as personal digital assistants and brochures) (57.7%), followed by time constraints (53.9%), a lack of library resources in Chinese (52.5%), deficient basic knowledge (48.8%), deficient skills in critical appraisal (48.4%), an insufficient number of capable designated personnel (46.2%), limited space for access to EBP resources (44.5%), deficient skills in literature searching (42.7%), insufficient library resources (38.8%), a lack of incorporation with clinical practice (30.6%), a lack of an EBP-supportive organizational climate (22.3%), and a lack of support from superiors (15.5%). There were significant differences in both personal and environmental barriers among the six groups of professionals. Physicians perceived fewer personal and environmental barriers than did those in other professional groups (p < 0.001).

**Table 2:** Barriers to evidence-based practice (EBP) implementation

**Lack of – N (%)**	**All**	**Physician**	**Nurse**	**Pharmacist**	**Physical therapist**	**Technician**	**Other**	**p - value**
	**N = 5,038**	**N = 614**	**N = 3,354**	**N = 401**	**N = 149**	**N = 382**	**N = 138**	
**Personal**								
Time, due to a heavy clinical load	2,714 (53.9)	253 (41.2)	1,859 (55.4)	233 (58.1)	90 (60.4)	207 (54.2)	72 (52.2)	<0.001
Basic knowledge	2,461 (48.8)	175 (28.5)	1,798 (53.6)	186 (46.4)	65 (43.6)	183 (47.9)	54 (39.1)	<0.001
Skills in critical appraisal	2,437 (48.4)	227 (37.0)	1,692 (50.4)	235 (58.6)	58 (38.9)	162 (42.4)	63 (45.7)	<0.001
Skills in literature searching	2,150 (42.7)	178 (29.0)	1,519 (45.3)	193 (48.1)	54 (36.2)	150 (39.3)	56 (40.6)	<0.001
Clinical incorporation	1,543 (30.6)	132 (21.5)	1,111 (33.1)	124 (30.9)	32 (21.5)	106 (27.7)	38 (27.5)	<0.001
**Environmental**								
Convenient application kits	2,905 (57.7)	312 (50.8)	1,916 (57.1)	256 (63.8)	97 (65.1)	244 (63.9)	80 (58.0)	<0.001
Library resources in Chinese	2,646 (52.5)	328 (53.4)	1,690 (50.4)	243 (60.6)	91 (61.1)	209 (54.7)	85 (61.6)	<0.001
Capable designated personnel	2,330 (46.2)	202 (32.9)	1,619 (48.3)	191 (47.6)	69 (46.3)	189 (49.5)	60 (43.5)	<0.001
Space for access to EBP resources	2,243 (44.5)	234 (38.1)	1,476 (44.0)	199 (49.6)	93 (62.4)	190 (49.7)	51 (37.0)	<0.001
Library resources	1,956 (38.8)	177 (28.8)	1,373 (40.9)	140 (34.9)	53 (35.6)	157 (41.1)	56 (40.6)	<0.001
An EBP-supportive organizational climate	1,122 (22.3)	107 (17.4)	808 (24.1)	81 (20.2)	28 (18.8)	71 (18.6)	27 (19.6)	0.001
Support from superiors	779 (15.5)	76 (12.4)	584 (17.4)	44 (11.0)	18 (12.1)	40 (10.5)	17 (12.3)	<0.001

In addition, 1,528 health professionals (30.3%) reported having participated in a training course for EBP implementation and/or use. Physicians and pharmacists reported more often participating in a training course for EBP than did the other professionals (p < 0.001) (data not shown).

### Factors associated with implementation of EBP

The univariate analyses showed significant correlations of self-reported EBP implementation with the following 13 characteristics: beliefs, attitudes, knowledge, skills, barriers, training, gender, a faculty position, a director position, academic degree, working experience, age, and one’s profession. Multivariate logistic regression analyses (Table 3) demonstrated that EBP implementation was more common in healthcare personnel with positive beliefs (OR = 1.49, 95% CI = 1.23 to approx. 1.79), favorable attitudes (OR = 1.25, 95% CI = 1.05 to approx. 1.48), sufficient knowledge (OR = 1.66, 95% CI = 1.38 to approx. 1.99), sufficient skills (OR = 1.35, 95% CI = 1.15 to approx. 1.59), participation in EBP training (OR = 1.91, 95% CI = 1.62 to approx. 2.26), and a faculty position (OR = 1.49, 95% CI = 1.25 to approx. 1.78). Healthcare personnel with a bachelor’s (OR = 1.24, 95% CI = 1.02 to approx. 1.50) or master’s or doctoral degree (OR = 1.45, 95% CI = 1.06 to approx. 1.98) were more likely to implement EBP than those who had graduated from a technical school. In addition, physicians (OR = 1.93, 95% CI = 1.39 to approx. 2.68) and other allied healthcare professionals (including speech language therapists, psychological consultants, respiratory therapists, and dietitians) (OR = 1.53, 95% CI = 1.01 to approx. 2.31) implemented EBP more frequently than nurses, whereas technicians were less likely to implement EBP than nurses (OR = 0.57, 95% CI = 0.44 to approx. 0.76). In contrast, EBP implementation was less common among healthcare professionals who reported a lack of basic knowledge (OR = 0.82, 95% CI = 0.69 to approx. 0.96) and skills in literature searching (OR = 0.78, 95% CI = 0.64 to approx. 0.95) as barriers to EBP use.

**Table 3:** Factors associated with the implementation of evidence-based practice (EBP), by a multivariate logistic regression analysis

**EBP implementation**	**Yes**	**No**	**OR**	**95% CI**	**p-value**
	**N = 2111**	**N = 2927**			
**Beliefs**	1,696 (80.3)	1,908 (65.2)	1.485	1.230 ~ 1.792	<0.001
**Attitudes**	1,476 (69.9)	1,539 (52.6)	1.248	1.052 ~ 1.479	0.011
**Knowledge**	788 (37.3)	656 (22.4)	1.655	1.377 ~ 1.989	<0.001
**Skills**	536 (25.4)	312 (10.7)	1.354	1.153 ~ 1.591	<0.001
**Training**	981 (56.5)	547 (18.7)	1.914	1.618 ~ 2.264	<0.001
**Barriers – lack of**					
Time	710 (33.6)	1,246 (42.6)	1.042	0.886 ~ 1.225	0.621
Basic knowledge	710 (33.6)	1,246 (42.6)	0.816	0.694 ~ 0.959	0.014
Skills in critical appraisal	710 (33.6)	1,246 (42.6)	1.013	0.837 ~ 1.226	0.895
Skills in literature searching	905 (42.9)	1,532 (52.3)	0.777	0.640 ~ 0.944	0.011
Clinical incorporation	1,188 (56.3)	1,717 (58.7)	0.844	0.708 ~ 1.006	0.059
Convenient application kits	1,045 (49.5)	1,669 (57.0)	1.132	0.950 ~ 1.349	0.167
Capable designated personnel	748 (35.4)	1,402 (47.9)	0.978	0.824 ~ 1.162	0.804
Space for access to EBP resources	858 (40.6)	16.3 (54.8)	0.990	0.830 ~ 1.182	0.914
Library resources	899 (42.6)	1,431 (48.9)	0.849	0.716 ~ 1.007	0.060
Organizational climate	519 (24.6)	1,024 (35.0)	1.043	0.832 ~ 1.307	0.716
Support from superiors	873 (41.4)	1,370 (46.8)	0.826	0.635 ~ 1.075	0.156
**Demographic**					
Male	495 (23.4)	320 (10.9)	1.124	0.868 ~ 1.454	0.375
Director	358 (17.0)	239 (8.2)	1.237	0.975 ~ 1.569	0.080
Faculty	633 (30.0)	496 (16.9)	1.490	1.247 ~ 1.780	<0.001
**Profession**					
Physician	436 (20.7)	178 (6.1)	1.932	1.394 ~ 2.678	<0.001
Pharmacist	169 (8.0)	232 (7.9)	0.801	0.610 ~ 1.051	0.110
Physical therapist	78 (3.7)	71 (2.5)	1.137	0.764 ~ 1.692	0.526
Technician	122 (5.8)	260 (8.8)	0.574	0.436 ~ 0.755	<0.001
Other allied	75 (3.6)	63 (2.2)	1.527	1.009 ~ 2.310	0.045
Nurse	1,231 (58.2)	2,123 (72.5)	reference		
**Working experience (years)**					
<5	630 (29.8)	981 (33.5)	0.937	0.727 ~ 1.208	0.617
5 ~ 10	782 (33.0)	1,152 (39.4)	0.928	0.762 ~ 1.130	0.457
>10	699 (33.2)	794 (27.1)	reference		
**Academic level**					
Master’s or doctorate	275 (13.0)	142 (4.8)	1.445	1.056 ~ 1.978	0.022
Bachelor’s	861 (40.8)	912 (31.2)	1.240	1.024 ~ 1.501	0.027
Junior college	521 (24.7)	943 (32.2)	1.078	0.904 ~ 1.285	0.403
Technical school	454 (21.5)	930 (31.8)	reference		
**Age (years)**					
20 ~ 30	775 (36.7)	1,308 (44.7)	1.331	0.985 ~ 1.799	0.063
31 ~ 40	974 (46.1)	1,237 (42.3)	1.214	0.961 ~ 1.534	0.104
>40	362 (17.2)	382 (13.0)	reference		

## Discussion

In this study, we describe perceptions of EBP implementation among medical, nursing, pharmacological, and allied healthcare professionals in Taiwan. Our results demonstrate that a majority of healthcare personnel have favorable beliefs in and attitudes toward EBP. However, their knowledge of and skills in EBP are limited. These findings are in accordance with previous studies of a variety of professional healthcare groups [10,13-17,27-29]. In addition, our data showed that EBP has not yet widely diffused into all groups of healthcare professionals in Taiwan. The results of this study have clinical implications for healthcare organization leaders and policymakers who wish to disseminate and implement EBP more broadly.

There were important differences related to EBP across the professional groups. Discrepancies among the groups in awareness of, beliefs in, attitudes toward, knowledge of, skills in, barriers to, and behavior regarding EBP were wide. First, physicians and pharmacists were the most aware of EBP. This is likely due to a long history of exposure to and efforts to influence the use of EBP [16]. In contrast, technicians were the least aware of EBP. A similar report from Upton *et al.* showed that technicians have poor awareness of EBP [12]. In addition, our results identified a number of factors related to an awareness of EBP, such as age and gender. Nevertheless, these factors were exclusively associated with the demographic characteristics of the professions [30]. Second, physicians and pharmacists were more likely to recognize the value of EBP. These findings are generally consistent with results of previous studies [13,17,27]. Burkiewicz *et al.* reported positive views toward EBP in as many as 90% of pharmacists [13]. Furthermore, nurses held the most unfavorable beliefs and attitudes toward EBP; this may have been due to unfamiliarity with EBP [16]. Third, physicians had more sufficient knowledge and skills than the other groups. This may be because EBP is a part of the initial educational training for physicians. Similar studies supported our findings in showing that physicians possess greater capability to implement EBP than nurses and allied healthcare personnel [16,17]. Fourth, physicians reported implementing EBP the most, whereas nurses and technicians implemented EBP the least. It is possible that clinical roles of physicians, nurses, and technicians are divergent. Physicians need to find evidence based on the best quality and make clinical decisions for the most effective healthcare. In contrast, hospital-based nurses and technicians may rely more on clear directions rather and may have less discretion in medical decision-making.

In addition to examining EBP-related differences among those in different professions, our study highlighted several factors in relation to implementing EBP. First, positive perceptions and high self-efficacy appear to be primary influences on implementation. Other studies similarly indicated that EBP users have more-favorable attitudes toward, knowledge of, and skills in EBP than non-users [23,31,32]. Second, a faculty position, educational training, and academic degree are important factors affecting implementation of EBP. Our previous study identified being a faculty member as a significant position from which to search for evidence-based information [33]. Furthermore, our data showed that healthcare professionals with a high academic degree or with educational training more often implemented EBP than those without either of these. Third, deficient knowledge and skills were negative predictors of EBP implementation. Taken together, our data support the importance of providing advanced education and training courses to facilitate the implementation of EBP. Our findings concur with other available literature showing that effective teaching programs can increase behaviors that support EBP implementation [2,34].

In terms of barriers to implementing EBP, the results of this study are similar to findings in other countries [15,20,35,36]. A lack of time is the most commonly reported personal barrier for healthcare professionals around the world [14,37-39]. In addition, insufficient knowledge and skills are significant barriers to EBP [37,40]. Nevertheless, our data indicated that a number of barriers were unique to organizational settings. In particular, our survey found that language barriers were a significant barrier to our participants. This is supported by our previous survey showing that nurses preferred evidence-based resources to be available in Chinese [26].

There are some limitations to this study. First, this was a self-administered survey, not an audit of actual practice; the results might not reflect the realities of practice under routine clinical care [22]. Second, inaccuracies may have occurred in the questionnaire survey; however, there is no other reliable method for collecting such data on a nationwide basis. Third, the return rate of this questionnaire survey was 59.1%; however, we believe our respondents are a representative sample because their backgrounds were similar to those in our previous surveys [16,26,30]. In spite of these limitations, our survey presents several potentially useful findings. Our study differs from previous studies examining information-searching patterns in that we evaluated self-reported EBP-related behaviors in the context of clinical decision-making, which is a vital component in implementing research evidence into clinical practice [22]. To our knowledge, this study is the first survey to systematically assess EBP implementation across physicians, nurses, pharmacists, and allied healthcare professionals.

## Conclusions

The significance of the present study stems from its focus on evaluating implementation of EBP in daily clinical practice. We used a large-scale questionnaire survey to compare various levels of perceptions toward EBP among all groups of healthcare professionals. Our data showed that healthcare professionals more often integrate evidence into clinical decision-making when the following characteristics are present: positive perceptions toward EBP, high self-efficacy to perform EBP, educational training for EBP, and having a faculty position and a high academic degree. To the extent possible, education and training that support these factors may help to increase positive beliefs and attitudes regarding EBP, and ultimately, EBP use in practice.

## Competing interests

The authors declare that they have no competing interests.

## Authors’ contributions

YHW, KNK, and YWC conceived and developed the study and drafted the manuscript. KNK and YWC were responsible for organizing the team and obtaining funding. CYY, HLL, and CC assisted with coordinating the study and drafting the manuscript. All authors read and approved the final manuscript.
